# How Inhomogeneous Site Percolation Works on Bethe Lattices: Theory and Application

**DOI:** 10.1038/srep22420

**Published:** 2016-03-01

**Authors:** Jingli Ren, Liying Zhang, Stefan Siegmund

**Affiliations:** 1 School of Mathematics and Statistics, Zhengzhou University, 450001 Zhengzhou, P.R. China; 2 Center for Dynamics & Institute for Analysis, Department of Mathematics, TU Dresden, 01062 Dresden, Germany

## Abstract

Inhomogeneous percolation, for its closer relationship with real-life, can be more useful and reasonable than homogeneous percolation to illustrate the critical phenomena and dynamical behaviour of complex networks. However, due to its intricacy, the theoretical framework of inhomogeneous percolation is far from being complete and many challenging problems are still open. In this paper, we first investigate inhomogeneous site percolation on Bethe Lattices with two occupation probabilities, and then extend the result to percolation with *m* occupation probabilities. The critical behaviour of this inhomogeneous percolation is shown clearly by formulating the percolation probability 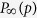 with given occupation probability *p*, the critical occupation probability 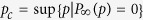, and the average cluster size 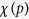 where *p* is subject to 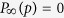. Moreover, using the above theory, we discuss in detail the diffusion behaviour of an infectious disease (SARS) and present specific disease-control strategies in consideration of groups with different infection probabilities.

Percolation (for reviews see Stauffer 1979^1^, Essam 1980^2^) is the random occupation of sites or bonds on lattices or networks, named as site percolation or bond percolation, respectively. Site percolation is more general than bond percolation because every bond model may be reformulated as a site model (on a different graph) and the converse is in general not true^3^. The appeal of percolation is the occurrence of a critical phenomenon, which has attracted attention for a wide range of applications: liquid flows in porous media^4^,^5^, epidemic spread^6^,^7^,^8^, granular and composite materials^9^,^10^,^11^,^12^, forest fires^13^,^14^,^15^ and fracture patterns and earthquakes in rocks^16^.

The research originated from homogeneous percolation, i.e., percolation with a single occupation probability. For example, Fisher and Essam (1961) solved homogeneous percolation problems on Bethe lattices^17^; Sykes and Essam (1964) studied the exact critical occupation probabilities in two dimensions^18^; Gerald (*et al.*, 2001) derived the value of critical occupation probabilities for a four-dimensional percolation problem on hyper-cubic lattices^19^.

Building on those results, researchers began to study inhomogeneous percolation^20^,^21^,^22^,^23^,^24^,^25^,^26^, in which sites (or bonds) may have different occupation probabilities. In 1982, Kesten^20^ obtained a critical surface of occupation probabilities for inhomogeneous percolation on square lattices. In 2013, Grimmett^21^ extended the result of inhomogeneous bond percolation to triangular and hexagonal lattices utilizing Russo-Seymour-Welsh (RSW) theory of box-crossings. Recently, Radicchi^27^ studied percolation on not necessarily infinite graphs and used graph decomposition methods to identify abrupt and continuous changes in percolation.

In the current paper, we focus on inhomogeneous percolation on Bethe lattices. The interest in a good understanding of this inhomogeneous percolation process is twofold. Firstly, Bethe lattices might hold fractal structures, which allow more complex behaviour than square or triangular lattices. The results of this inhomogeneous percolation on Bethe lattices have more extensive applications, e.g., the spreading problem of social networks or infectious diseases (see section III). Secondly, as is well known, most results on percolation are obtained using the heuristic approximation or numerical approaches^27^,^28^,^29^ and it is difficult to get the exact solutions for average cluster sizes and the percolation probability even for homogeneous percolation. In this paper, we investigate inhomogeneous site percolation on Bethe lattices from two occupation probabilities to *m* occupation probabilities: for the case of two occupation probabilities, we present the explicit formula of the critical occupation probability and the exact solution of average cluster size 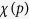; and for the case of *m* occupation probabilities, because of computational complexity, we formulate and obtain the numerical solutions of average cluster size and percolation probability, which might shed light on deriving the results of inhomogeneous percolation. Besides, we analyse in detail the spread of SARS (an infectious disease) using this inhomogeneous percolation on Bethe lattices with dynamically changing parameters. We present specific control strategies for SARS by comparing the critical infection probabilities, the average numbers of infected individuals for each day, and the probability of the large-scale outbreak of SARS.

## Basic theory

In ref. 30 a Bethe lattices is defined to be a tree where each site has *Z* neighbours, *Z* is also named coordination number. For the sake of convenience, we denote it as 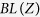.

**Figure 1:**
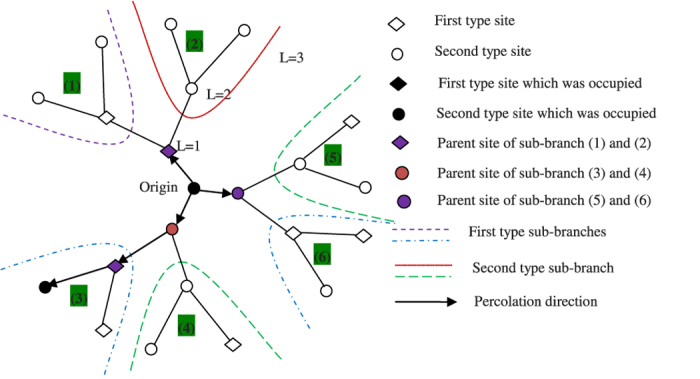
*PBL*(3; (1, *p*_1_), (2, *p*_2_)) with 3 sub-generations.

On a Bethe lattices, if all sites are occupied randomly with the same probability *p*, independent of its neighbours, we call the percolation process as homogeneous site percolation and write it as 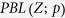. If the sites of a Bethe lattices 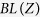 are occupied with different probabilities, then the percolation is inhomogeneous. To be concrete, without loss of generality, assume that *Z* neighbours of each site are occupied randomly with *m* 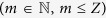 occupation probabilities 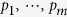, and then, we divide *Z* neighbours of each site into *m*different groups (*m* is the number of types of neighbours according to occupation probabilities), where, 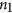 of *Z* neighbours are sites with occupation probability 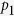, 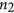 are sites with occupation probability 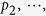 and 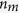 are sites with occupation probability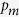. The inhomogeneous percolation is indicated with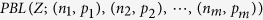. See Fig. 1 for an illustration of  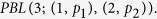


In this paper, we first consider inhomogeneous percolation on a Bethe lattices with 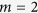, that is 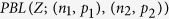. Clearly, in this case if 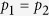 or 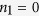 or 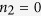, inhomogeneous percolation will specialize to homogeneous percolation on *BL(Z)*^30^. In a second step, we generalize the results of 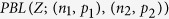 to the case of 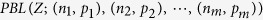.

### Critical surface of occupation probability

#### 

**Figure 2:**
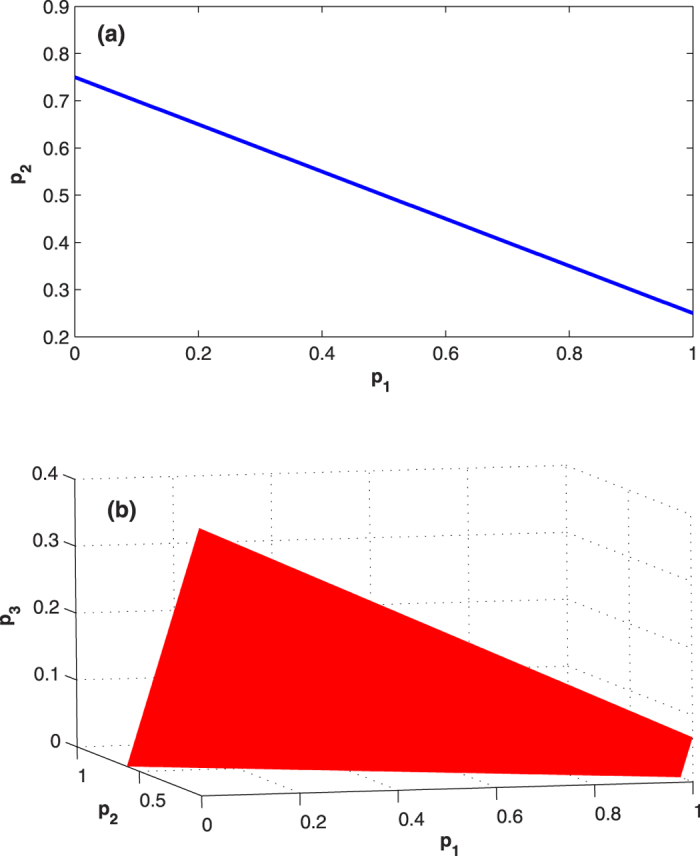
Critical surface of occupation probability. (**a**) Critical surface of occupation probability of 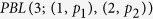. (**b**) Critical surface of occupation probability of 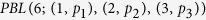.

Occupation probability is the probability with which the sites of a network are occupied. For 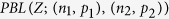, assume that a grandparent site is a first type-site on an infinite lattice, then for the parent site, there are 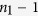 sub-branches that begin with the first type-sites and 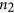 sub-branches beginning with the second type-sites (Fig. 1.). According to the binomial distribution, only 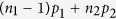 branches are accessible on average. On the other hand, if the grandparent site is a second type-site, then for the parent site, there are 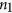 sub-branches that begin with first type-sites and 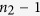 sub-branches which begin with second type-sites. In this case, on average, only 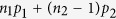 branches are accessible. Recalling that the ratio of these two types of sites is 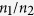, and according to expectation theory, on average, only


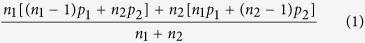


branches are accessible at each step. In order to get an infinite cluster, it is necessary that the quantity (1) is equal or greater than one. Therefore, the critical condition such that an infinite cluster (percolating cluster) first occurs is


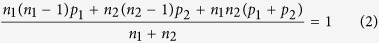


from equation (2), we derive the critical surface of occupation probability


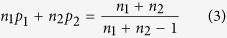


this critical surface of 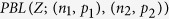 is a line with slope 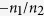 in the occupation probabilities set 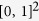. As an example, see Fig. 2(a) for an illustration of the critical surface of 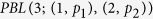.

In a similar way, we can derive the following result.

**Theorem 1**. The critical surface of 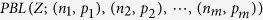 is given by


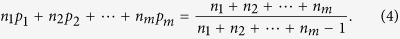


### Proof of Theorem 1

For 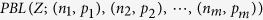, if the grandparent site is an *i*th 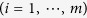 type-site, then for the parent site, there are 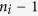 sub-branches that begin with an *i*th type-site and *n*_*k*_ 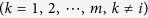 sub-branches beginning with *k*th type-sites (Fig. 1). According to the multi-binomial distribution, in this case only 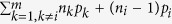 branches are accessible on average.

Recalling the occupation probabilities for all types of sites and according to expectation theory, overall, only


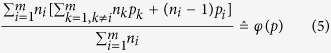


branches are accessible on average. In view that 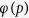 is equal to one on the critical surfaces, we have equation (4), the critical surface of 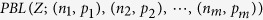.

**Figure 3:**
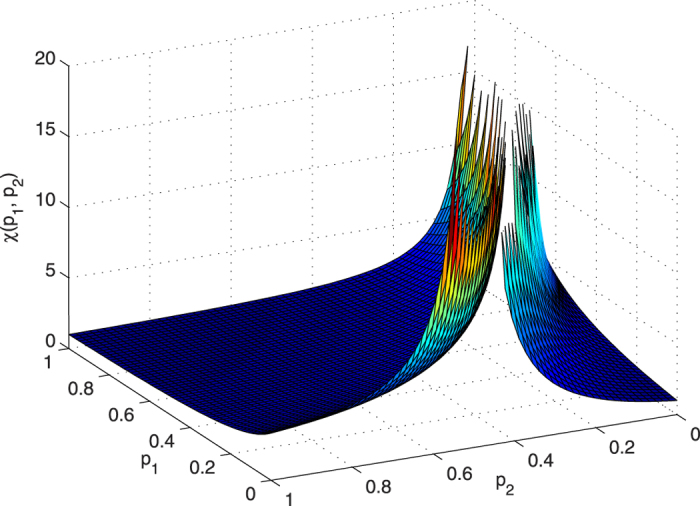
Average cluster size *χ*(*p*) of *PBL*(3; (1, *p*_1_), (2, *p*_2_)). The left-hand curve is a sketch of the average cluster size 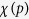 and the right-hand curve is a sketch of the mean size of finite clusters of occupied sites when 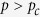.

It is clear that 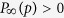 if 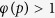; 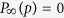 if 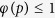, where, 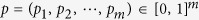; 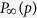 is the percolation probability (existing infinite clusters). The theory generalizes the concept of exact critical value 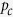 in homogeneous percolation. For consistency, we also indicated the critical surface of 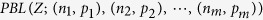 by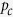. The critical surface 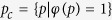 is the subset of the occupation-probability set 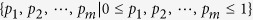 with all 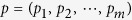 satisfying equation (4). Then, in the supercritical phase, 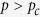 stands for 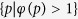, and there exists almost surely at least one infinite cluster of occupied sites. Contrarily, in the subcritical phase, 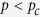 stands for 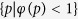, and all clusters of occupied sites are almost surely finite. For an illustration, Fig. 3(b) is the critical surface of 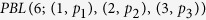.

For an intuitive understanding, take 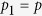 as a reference, and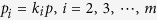, we have from equation (4) that 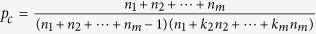. In this case 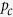 is degenerated to a critical point, which is consistent with homogeneous percolation (in the supercritical phase, 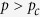 and in the subcritical phase, 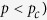.

The critical surface of occupation probabilities consists of those probabilities at which the percolation behaviour of the system changes essentially. We can control the percolation behaviour if we know the critical surface of occupation probabilities.

#### Average cluster size of occupied sites

The average cluster size 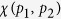 of occupied sites is the mean size of the finite (non-percolating) clusters of occupied sites. It is closely related to the critical surface of occupation probabilities. We consider 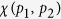 and the relationship between 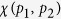 and critical surface.

### Theorem 2

Assume that the Bethe lattice 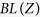 is infinite with occupation probabilities 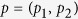 such that 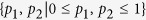, then the average cluster size of occupied sites of 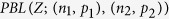 satisfies


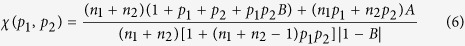


where 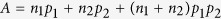, 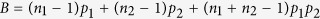.

**Proof of Theorem 2**. If the Bethe lattice is infinite, all sites are equivalent for evaluating the average cluster size of occupied sites. Let 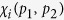 be the average cluster size of the centre sites which are of type *i* 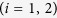, and 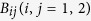 be the contribution (to the average cluster size) from a sub-branch which begins with a *j*th type-site and whose parent site is an *i*th type-site (Fig. 1). Then


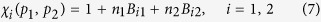


where, the first term is the contribution from the centre site itself, the second term is the contribution from the first type branches, and the third term is the contribution of the second type branches.

According to expectation theory, on average, the average cluster size is


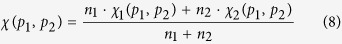


Based on definition of inhomogeneous percolation on a Bethe lattice, the following recurrence relations can be concluded


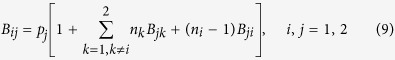


Solving 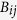 from equation (9), we have from (7) and (8) that


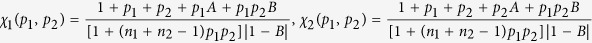


and


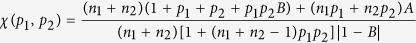


where, 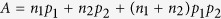 and 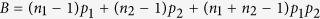.

For 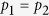 or 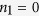 or 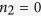, equation (6) reduces to 
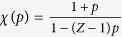
.

The average cluster size (of occupied sites) 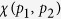, which is a function of the occupation probability 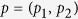, can reveal the intensity of percolation. For 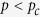, 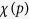 increases rapidly with *p* (Fig. 3), and diverges in a power law of the distance between *p* and *p*_*c*_, as *p* approaches *p*_*c*_ from below. For 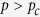, there exist infinite clusters of occupied sites and their number increases as 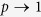. On the other hand, the numbers of finite clusters (of occupied sites) and their sizes are reducing. Therefore, for 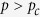, the average sizes of finite clusters 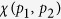 decrease with *p* increasing (Fig. 3).

Similarly, generalizing the result to 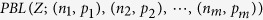, we first solve 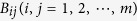 from equation (10), where the 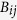 have the same meaning as above


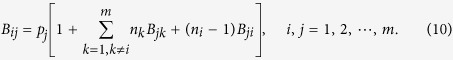


Then, substitute the solution of equation (10) into equation (11) to get the values of 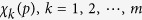, here 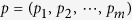.


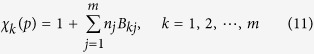


According to expectation theory, the average cluster size is


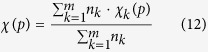


Because the explicit expression of 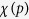 is very complex, we provide only the derivation process.

### Percolation probability

**Figure 4:**
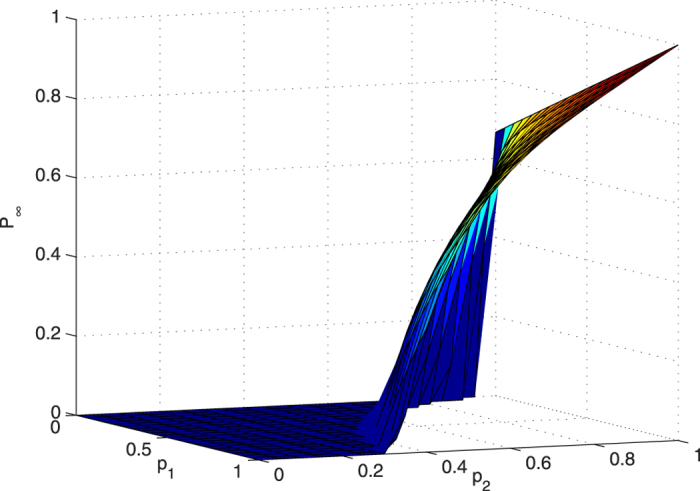
Percolation probability *P*_∞_(*p*) of *PBL* (3; (1, *p*_1_), (2, *p*_2_)).

In this part, we mainly discuss the percolation probability, i.e., the probability that the origin site belongs to a percolating infinite cluster. Percolation probability is indicated as 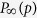 that can reveal the intensity of percolating, for 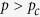 (Fig. 4).

For 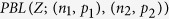, in order to determine 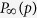, let 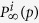 denote the probability that an *i*th type origin site belongs to a percolating infinite cluster, where 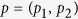, 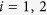.

A site belongs to a percolating infinite cluster, which means, not only the site itself is occupied, but also at least one of the 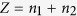 branches (originating from the site) connects to the percolating cluster. Both of these are independent of each other, so, 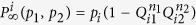. According to mean theory, it can be concluded that


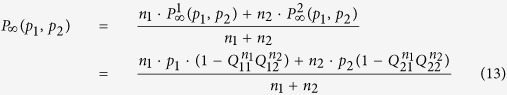


where, 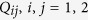, is the probability that a sub-branch does not connect to the percolating cluster and the sub-branch begins with a *j*th type-site, whose parent site is an *i*th type-site. Then, we have


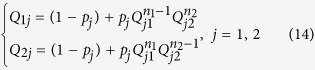


for each equation of (14), the first term is the probability that the root site of a sub-branch is not occupied and the second term is the probability that the root site of the sub-branch is occupied but no child sub-branch connects to the percolating cluster.

If 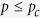, equation (14) has only the trivial solution 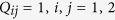, then from (13) we have 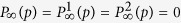 (Fig. 4). If 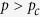, there is a nontrivial solution for equation (14) and then equation (13) has a nonzero solution. In this case, it is not easy to get the exact nontrivial solution of equation (14) for it is a set of multivariable high-order equations. By employing fixed-point iteration, we get a numerical solution of equation (14) instead and then obtain the percolation probability from (13). From the numerical solution (Fig. 4), it can be seen that 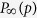 picks up abruptly at *p*_*c*_ then increases rapidly with *p* increasing.

We extend the result to 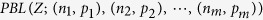 by a similar analysis. In this general case, 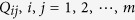 satisfy 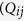 meaning the same as above)


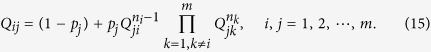


Firstly, derive the solution of (15) by fixed-point iteration, and then substitute 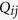 into (16),


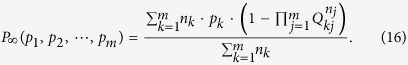


This way, we get 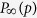, where 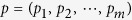.

### Percolation model of the disease spreading (SARS in Beijing, 2003)

Severe acute respiratory syndrome (SARS) is a viral respiratory illness caused by a corona-virus. In Beijing (China), about 2523 cases have been infected with SARS in 2003. At the beginning of emergence, because of the lack of understanding of SARS and the high mobility of the modern-social activities, SARS spread rapidly. Afterwards, when people found the high infectivity and death rate of SARS, they begun to limit social activity of the public and take strict isolated measure to prevent the spreading of disease then the disease was contained. Is this the proper infection control measure of SARS? Which kind of infectious diseases are suitable for this approach?

In fact, the spreading of SARS is a percolation process. Considering the differences of physical resistibility or intimate contact with the infected individual, we divided people into two groups: the people with higher infection probability (e.g., infants and elderly or healthcare workers), named as susceptible persons; and the people with lower infection probability, named as common persons. Then this disease is modelled as inhomogeneous percolation on Bethe lattices with two occupied probabilities, i.e., 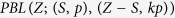. Here, *Z* is the average contact number of each person, *S* (of *Z*) denotes susceptible persons with infection probability *p* and *Z-S* (of *Z*) is common persons with infection probability *kp* 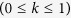.

The first case of SARS was confirmed on March 5 (in Beijing, 2003) and the government started reporting the cases from April 20. According to case-reporting data from April 20 to June 23 (in Beijing, 2003) (from the government bulletin) and the control activities of government, we find three other critical time points of SARS: May 1 (it is legal holidays from May 1 to May 7), May 14 (people generally panicked over SARS and limited their social activities), and May 30 (new cases of SARS considerably decreased). Correspondingly, the spreading process of SARS was divided into five stages. Then, by random simulation, we found that the spreading of SARS has a fifteen-day time delay. Therefore, we changed the five stages of the spreading process of SARS into: stage 1—from March 20 to May 4, stage 2—from May 5 to May 15, stage 3—from May 16 to May 28, stage 4—from May 29 to June 13, and stage 5—from June 14 to June 23.

In the initial stage, the average contact number of one infected patient was around fifteen 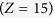, in which two or three person had infected SARS (Gong *et al.*^31^), so the infection probability was around 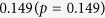. We take 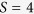 according to the percentage of susceptible people in the population and we set 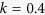 by statistical investigation. It is worth noting that the infection probability *p* is the manifestation of the spreading intensity of the disease, which can only be slightly affected by the protective approach. Therefore, the infection probability was adjusted to 0.1425 in the second stage and third stage, and was adjusted to 0.141 in the fourth stage and fifth stage. The other parameters, i.e., *Z* and *S* would change with prevention (isolation of infected patient and restriction of travel) and *k* remains unchanged in different stages. By statistical investigation, we set *Z* = 13 and *S* = 4 in second stage, *Z* = 12 and *S* = 3 in third stage, *Z* = 11 and *S* = 3 in fourth stage, *Z* = 8 and *S* = 2 in fifth stage.

Obviously, the spreading model of SARS (in Beijing) is inhomogeneous percolation on a Bethe lattice with dynamically changing parameters. See Table 1 for the model division and the parameters.

### Results of percolation model and control measures

From equation (3), equation (6), and equation (13), we acquire the critical infection probabilities, average number of infected individuals, and the probability of large-scale outbreak for the SARS percolation model 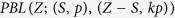 in different spreading stages (Table 1).

It can be concluded from Table 1, that, if one is infected with SARS and would live as a normal person, then the disease would infect a massive crowd of healthy people except for 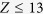 and 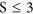. That warns us, at the initial stage of SARS, humankind should try their best to find SARS patients as early as possible and isolate them from healthy people. Nevertheless, during the incubation period, it is inevitable that some infected persons, who cannot be found and live as the normal person around us that is quite dangerous. At this time, the most effective way is to reduce outdoor activities of public then cut down the average contact number.

**Figure 5:**
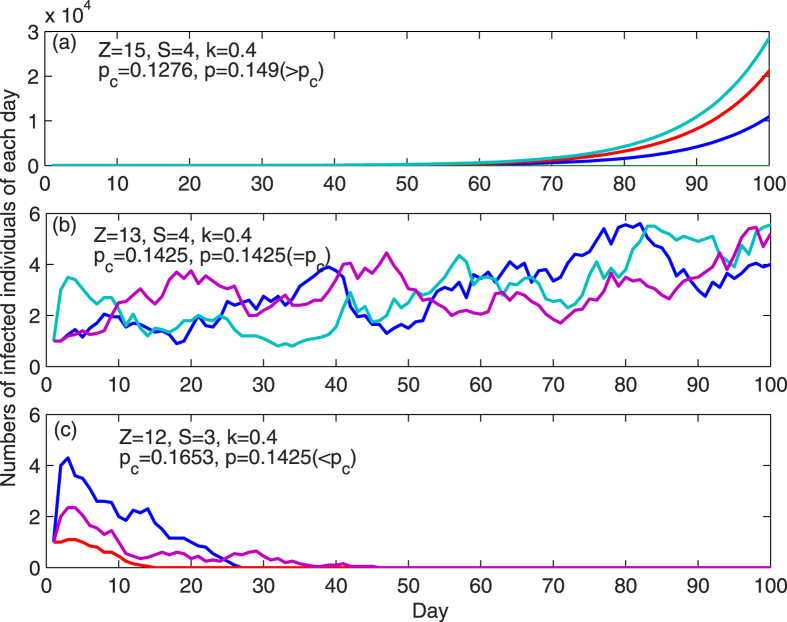
The average number of infected individuals versus every day with infection probabilities that are larger, equal, and smaller than critical infection probability.

For more accurate and meticulous disease-control strategies, we scrutinized the dynamic-dependent relationship between 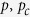 and the average number of infected cases with subtle parameters by a Monte-Carlo simulation. In the initial stage of the SARS process, from March 20 to April 8, according to the above analysis, 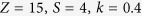, and 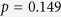, based on these, the average number of infected cases of each day was simulated. See Fig. 5(a) for an illustration. It is clear that the random variation of the infected number has an incremental trend and that implies that the disease would infect a large amount of persons. Then from April 9 to April 19, during the second stage of SARS, some protective measures were taken with the understanding of the disease, so parameters changed to 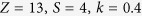, and 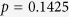. By simulation, we found that the infected number changes chaotically as in Fig. 5(b). With the time going by, from April 20 to May 4, the severity of the disease gradually being known, more protective measures were taken and parameters reduced further to 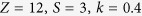, and 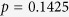. In this stage, the simulation revealed that the average infected number of each day fluctuates with a trend of decline and it would be zero after a period, which predicates the infectious disease can be controlled without any additional measures (Fig. 5(c)). In fact, by simulating the percolation, we acquire 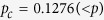 in the initial stage, 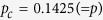 in the second stage, and 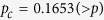 in the third stage, i.e., the initial stage is a supercritical phase of the spread of SARS, the second stage is a critical phase, and the third stage is a subcritical phase, which agrees with the simulation. We could conclude that, near the critical point, slightly adjusting of the system parameters would cause a fundamental change of the trend of infectious diseases. Therefore, in order control the large-scale outbreak of the disease, we must try our best to make 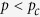, even if the infection probability is only a little smaller than the critical infection probability. Actually, if 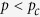, the probability of a large outbreak of SARS is zero; and if *p* reaches and crosses 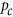, the probability picks up as a power law with exponent one in terms of *p-p*_*c*_, and the disease will outbreak rapidly.

As we know, reducing outdoor activities of the public is a powerful strategy for infectious disease with latent period but it severely obstructs the people’s daily life and social economy. The methods in this paper will supply a quantitative measure for the risk of disease outbreaks and to guide our practice more appropriately.

## Predictions

**Figure 6:**
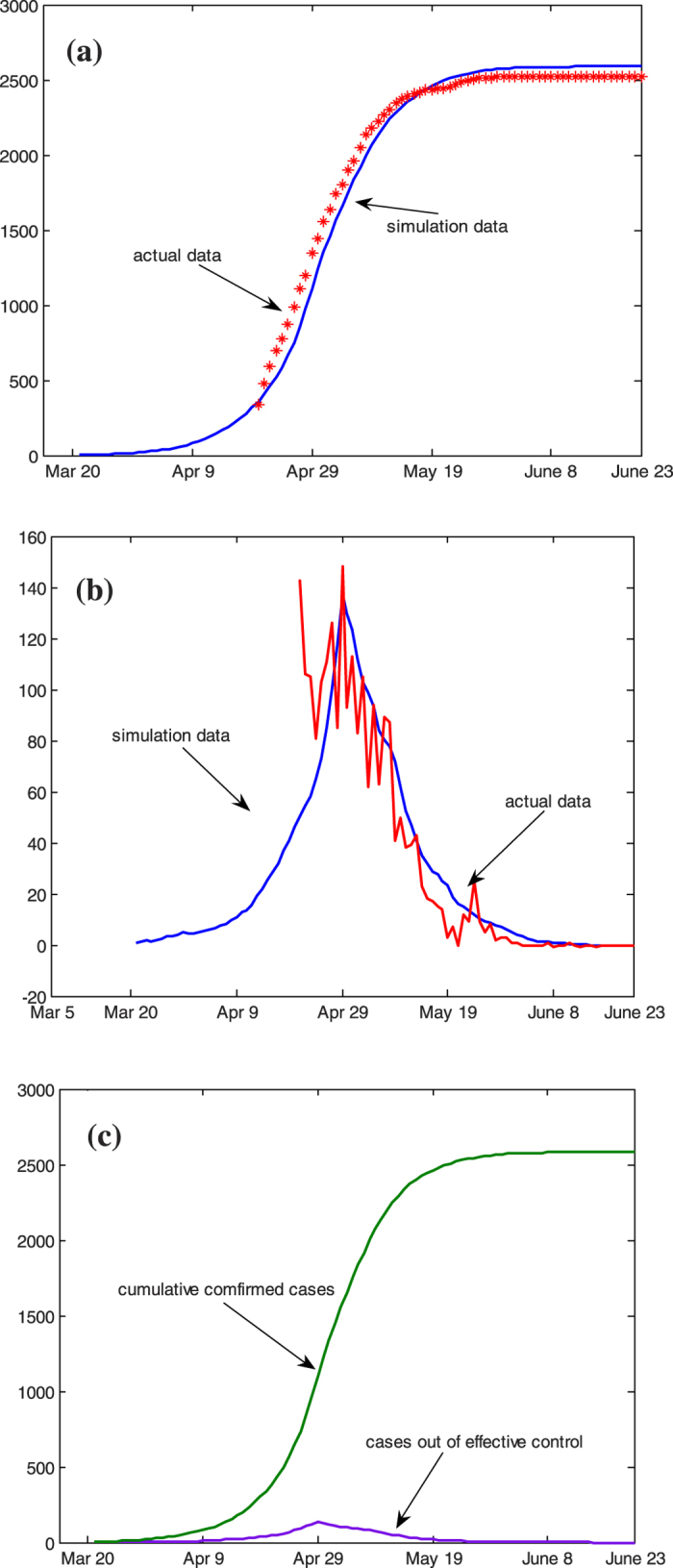
Simulation results (**a**) Cumulative cases of SARS with simulation data and actual data. (**b**) Daily new cases of simulation and report. (**c**) The cumulative cases and daily new cases by simulation.

Based on the model of the spreading of SARS, we could supplement the data of cases from March 5 to April 20, during which the recording data was missing. See Fig. 6.

Figure 6(a) is the actual cumulative case-reporting data and simulative data. New cases of each day are displayed in Fig. 6(b). We can find that the inhomogeneous percolation is in good fitting with the dynamic process of SARS spreading and time delay is the considerable feature of SARS. The relationship between cumulative confirmed cases and cases out of effective control displays in Fig. 6(c). It can be seen that there will be many persons infected with SARS even if only a few cases of SARS are out of effective control.

### Sensitivity analysis

The sensitivity index is the ratio of the change in output to the change in input of parameters or variables^32^. Taking into account the characteristics of the model, we employ a one factor at a time (OAT) approach, which is more agile and easy to interpret. The popular sensitivity index of OAT approach is 
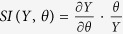
, where *Y* is the output, *θ* is the input, 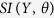 is the sensitivity index of *Y* to *θ*, and 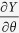 is the partial derivative of *Y* with respect to *θ*. The quotient 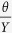 is introduced to normalize the index by removing the affects of units^33^.

First, we get 
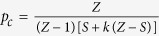
 from equation (3), and then derive that:


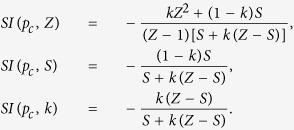


Where, 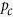 is the critical infection probability, one of the output of the SARS-percolation model; 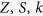 denote the average contact number of each patient, the number of susceptible persons of *Z*, the ratio of infection probabilities of susceptible person to common person (see page 12), respectively. They are all input parameters.

**Figure 7:**
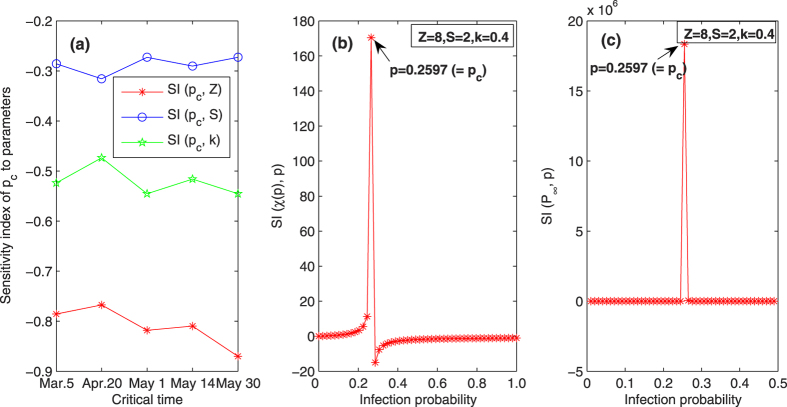
Sensitivity index (**a**) Sensitivity index of critical infection probability to input parameters-*Z*, *S* and *k* on five critical days of SARS in Beijing (2003) (**b**) Sensitivity index of the average infected cases by one patient to input variable *p* (infection probability). (**c**) Sensitivity index of the probability of SARS large-scale outbreak to input variable *p*.

Based on the Table 1, the sensitivity indexes of 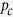
to three input parameters at five critical time points are obtained, which are all negative scalars. These suggest that the decreasing of each input parameters correspond to the increasing of 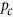. Among them, the absolute value of 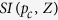 is about 0.8, which is maximum, 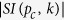 is about 0.5, and 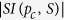 is about 0.3. It is clear that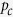 has greater sensitivity to *Z*. See Fig. 7(a) for an illustration.

By a similar analysis with a numerical approach, we obtain 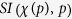 and 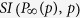. The sensitivity index 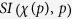 is shown in Fig. 7(b). It indicates that 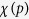 is more sensitive near the 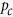. Since 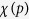 is the average size of all-finite clusters of infected cases, the 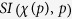 exhibits negative value when *p* is greater than 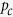. By a numerical approach, the sensitivity of the probability of large-scalar outbreak of SARS 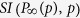 is displayed in Fig. 7(c). 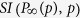 has similar characteristics as 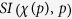. They are both more sensitive near the 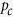.

## Conclusion

In this paper, we present a theoretical framework for inhomogeneous site percolation on Bethe Lattices, and apply it to investigate a diffusion problem of an infectious disease. It is found that the inhomogeneous percolation on Bethe lattices serves as an appropriate model to describe the dynamic spreading behaviour of the infectious disease (SARS). The percolation model of SARS is not only in good agreement with the actual recorded data, but also can be used to predict the future trend of the disease and supply the missing data of the past. Moreover, it can provide quantitative results for government to make more proper disease-control strategies.

## Additional Information

**How to cite this article**: Ren, J. *et al.* How Inhomogeneous Site Percolation Works on Bethe Lattices: Theory and Application. *Sci. Rep.* **6**, 22420; doi: 10.1038/srep22420 (2016).

## Figures and Tables

**Table 1:** The results of inhomogeneous percolation of SARS and some parameters by simulation and statistical analysis.

	Mar. 20-May 4	May 5-May 15	May 16-May 28	May 29-June 13	June 14-June 23
Infection probability—*p*	0.149	0.1425	0.1425	0.141	0.141
Average contact number—*Z*(*S, Z-S*)	15(4, 11)	13(4, 9)	12(3, 9)	11(3, 8)	8(2, 6)
Critical infection probability—*p*_*c*_	0.1276	0.1425	0.1653	0.1774	0.2597
Average infected persons by one patient	infinite	infinite	74.77	17.4	3.0277
Probability of large-scale outbreak—*p*_∞_	0.0233	0.0045	0	0	0
